# General Population Knowledge about Extreme Heat: A Cross-Sectional Survey in Lisbon and Madrid

**DOI:** 10.3390/ijerph14020122

**Published:** 2017-01-28

**Authors:** Julita Gil Cuesta, Joris Adriaan Frank van Loenhout, Maria da Conceição Colaço, Debarati Guha-Sapir

**Affiliations:** 1 Centre for Research on the Epidemiology of Disasters (CRED), Institute of Health and Society, Université Catholique de Louvain, Brussels 1348, Belgium; joris.vanloenhout@uclouvain.be (J.A.F.v.L.); debarati.guha@uclouvain.be (D.G.-S.); 2 Centre for Applied Ecology “Prof. Baeta Neves”/InBIO, Research Network in Biodiversity and Evolutionary Biology (CEABN/InBIO), Instituto Superior de Agronomia, University of Lisbon, Lisbon 1649-004, Portugal; ccolaco@isa.ulisboa.pt

**Keywords:** extreme weather events, population health, public health policy and practice

## Abstract

Extreme heat is associated with an increased mortality and morbidity. National heat plans have been implemented to minimize the effect of extreme heat. The population’s awareness and knowledge of national heat plans and extreme heat is essential to improve the community’s behavior and adaptation. A general population survey was conducted in Lisbon and in Madrid to assess this knowledge. We used a questionnaire to interview passers-by. Results were compared between Lisbon and Madrid and between locals and foreigners, using Pearson Chi-square tests and Fisher's exact test. We conducted 260 interviews in six locations of different socio-economic backgrounds in each city. The most frequently mentioned extreme heat-related risk groups were the elderly (79.2%), children (49.6%) and babies (21.5%). The most frequently reported protective measures were increased fluid intake (73.1%) and avoiding exposure to the sun (50.8%). Knowledge about the heat plan was higher in Lisbon (37.2%) than in Madrid (25.2%) (*p*-value = 0.03). Foreigners had less knowledge of risk groups compared to locals. Heat plans were not widely known in Madrid and Lisbon. Nonetheless, knowledge of practical concepts to face extreme heat, such as certain risk groups and protective measures, was found. Our results were similar to comparable surveys where specific respondents’ groups were identified as less knowledgeable. This highlighted the importance of addressing these groups when communicating public health messages on heat. Foreigners should be specifically targeted to increase their awareness.

## 1. Introduction

An increase in the number of unusually warm days has been observed at the global scale since 1950 [1,2]. In that context, the relationship between extreme heat and mortality has been widely documented [3,4,5,6,7]. There is also evidence for a relationship between extreme heat and morbidity [8,9,10].

The impact of extreme heat on mortality is influenced by the existence and quality of health services, living conditions such as housing, individual coping strategies and the existence of heat plans [11,12]. Consequently, national action plans have been implemented to prevent and minimize the impact of extreme heat on health in affected countries such as Portugal and Spain [13,14].

Heat-related risk perceptions can change according to personal experience and level of trust in authorities [15]. Perceptions of extreme heat-related risks may influence community behavior [16,17]. 

It is therefore important to understand the general population’s knowledge about national heat plans, extreme heat-related risk groups, symptoms and protective measures. We conducted a survey with residents in Lisbon and Madrid in order to assess that knowledge.

## 2. Materials and Methods

We carried out a cross-sectional observational study, using a questionnaire previously utilized in Brussels and Amsterdam (see Appendix A) [18]. We conducted the survey in Lisbon on 20–22 June 2016 and in Madrid on 29 June–1 July 2016. This summer period was chosen to be able to ask about heat awareness during a warm time. A team of two surveyors and a field coordinator carried out the survey in each city. We used a convenience sample of passers-by located in six survey locations in each city presenting different socio-economic backgrounds [19]. At least 20 interviews were carried out per each of the 12 survey locations. Passers-by were asked to participate and provided verbal consent. Interviews were conducted in Portuguese, Spanish or English. Individuals older than 12 years, living in Lisbon or Madrid and speaking one of the survey languages were eligible for inclusion. We excluded respondents who were not living in Lisbon or Madrid and without information on age, education or nationality.

The questionnaire included questions on the demographics age, sex, nationality and mother tongue. Locals or foreigners were categorized using nationality; locals were identified if respondents’ nationality was Portuguese in Lisbon or Spanish in Madrid, and foreigners if not. Mother tongue was established according to the nationality of the respondent. We used open questions to ask whether respondents could name any heat-related risk groups, symptoms for extreme heat, protective measures against adverse heat-related health effects and responsibility to take actions about increases in temperature, without providing the respondents with any answers. Multiple answers were possible and we recorded each of the reported answers of the open questions in a check-box format. We calculated the proportion of respondents who reported responses to these open questions. In addition, respondents were asked whether they were familiar with the national heat plan, whether they believed there has been an increase in temperature in the recent past, whether they considered themselves sensitive to extreme heat and whether the government was doing enough to raise awareness on this issue.

We compared the 12 survey locations that we had selected according to socio-economic background. We compared the results between Lisbon and Madrid, using Pearson Chi-square Tests and Fisher’s exact test when appropriate. A *p*-value (*p*) of <0.05 was considered to be statistically significant based on two-sided tests. Data were analyzed using Stata 14 (StataCorp, College Station, TX, USA).

Following the Portuguese and Spanish pertinent laws [20,21], the study was exempted from ethical committee approval, since data related to individual health was not collected, nor were individual names.

## 3. Results

### 3.1. Demographic Characteristics of Respondents

We conducted 260 interviews (129 in Lisbon and 131 in Madrid). There were no differences in age or sex between Lisbon and Madrid (Table 1). In Lisbon, 12.4% of the respondents were foreigners whereas 26.7% were in Madrid (*p* < 0.01) (Table 1). Forty-one percent of the respondents’ highest educational level was high school, and 42.7% had a university degree. Foreigners had statistically significant higher educational level than locals.

The survey locations showed a proportion of males which ranged from 33% to 76% in the six locations in Lisbon and from 38% to 64% in the six locations in Madrid. In Lisbon, the mean age among the six locations ranged from 40 years old (standard deviation (SD) 13) to 55 (SD 22) years old and in Madrid from 37 (SD 13) to 63 (SD 25) years old. The proportion of local respondents varied from 62% to 100% in the six locations in Lisbon, whereas it varied from 45% to 94% in Madrid.

### 3.2. Knowledge about Risk Groups, Symptoms, Protective Measures, Heat Plan and Government-Raised Awareness

With respect to heat-related risks, the elderly were recognized by the majority of respondents (79.2%) (Table 2). Nearly half of the respondents also recognized children as vulnerable to heat (49.6%), but only one-fifth specifically mentioned babies (21.5%) as a susceptible group. Respondents from Lisbon more often mentioned the risk for infants, medically ill individuals and individuals with social problems as compared to Madrid (Table 2).

Respondents were less knowledgeable on symptoms associated with extreme heat as compared to the recognized protective measures. The more frequently reported symptoms were dehydration (33.1% of the respondents), fatigue (21.9%) and dizziness (18.1%). The main protective measures were increasing fluid intake (73.1%), avoiding exposure to the sun (50.8%), using appropriate clothing (26.9%) and visiting green areas (26.1%). In Lisbon, respondents had a significantly higher understanding of certain heat-related symptoms such as nausea, dehydration and low blood pressure compared to Madrid (Table 2).

Thirty-one percent of the respondents had some general knowledge about the heat plan in their country of residence (37.2% in Lisbon and 25.2% in Madrid (*p* = 0.03)). The knowledge about the plan increased with age and educational level in Lisbon and Madrid, although not significantly. There was no difference in knowledge between men and women. Among the respondents who knew about the plan, the vast majority (79.7%) learned it through television and 10.1% through the Internet or social media. Only 4.5% of the persons familiar with the plan knew it had been activated last year (Table 2).

Most of the respondents (55.8%) thought that their national government raised too little awareness about extreme heat, but 40.0% found it to be adequate. In Lisbon, a higher proportion responded that the awareness was too little (64.3%) compared to Madrid (47.3%) (*p* = 0.02) (Table 2).

### 3.3. Personal Susceptibility, Increase in Temperature and Responsibilities

More than half of the respondents considered themselves not or only slightly sensitive to extreme heat and no difference was observed between Lisbon and Madrid. A majority (74.1%) believed there has been an increase in temperature in the recent past (lower in Lisbon (66.7%) than in Madrid (81.5%) (*p* = 0.02)). When respondents were asked who was responsible for taking actions against this increase, 66.7% considered the national government responsible, whereas 22.5% referred to citizens, 19.7% to the municipality, 12.8% to private companies and 8.1% believed everybody was responsible (Table 3).

### 3.4. Nationality and Knowledge about Heat

Eighty percent of the respondents were locals (87.6% in Lisbon and 73.3% in Madrid). Locals were significantly older and had a lower educational level in comparison with foreigners (Table 4).

Despite having lower educational levels, locals were significantly more knowledgeable about certain extreme heat-related risk groups compared to foreigners. These risks groups were the elderly (82.3% recognized by locals vs. 66.7% by foreigners (*p* = 0.01)), and babies (24.4% vs. 9.8% (*p* = 0.02)). Locals were also more aware of symptoms such as dehydration (35.4% of locals vs. 23.5% of foreigners (*p* = 0.1)) and fatigue (23.4% vs. 15.7% (*p* = 0.23)) and about protective measures such as avoiding exposure to the sun (52.6% of locals vs. 43.1% of foreigners (*p* = 0.22)), and about the existence of the heat plan in general (32.0% vs. 27.4% (*p* = 0.52)); these results were not significant. Out of the foreigners (*n* = 52), respondents whose mother tongue was Portuguese in Lisbon and Spanish in Madrid had a higher knowledge about the existence of the heat plan (42.3%) than foreigners with another mother language (12.0%) (*p* = 0.01). 

Locals believed more frequently that the government was responsible for the increase of the temperature compared to foreigners (70.0% vs. 52.9% (*p* = 0.03)). They felt more often that the government raised too little awareness (57.9% vs. 47.1%, (*p* = 0.2)). Locals also thought less frequently that average temperatures had increased compared to foreigners (70.6% vs. 88.2% (*p* = 0.03)). These differences in nationality did not change when stratified by educational level.

## 4. Discussion

Our study showed that less than a third of the respondents had knowledge about the existence of a national heat plan, whereas practical concepts to face extreme heat, such as knowledge of risk groups and protective measures, were widely recognized.

Regular exposure to extreme heat in Lisbon and Madrid can explain the knowledge of some of the practical measures to be protected against the heat. Also, the low perception of being sensitive to heat can be explained by the regular exposure to heat. Previous experience from a natural hazard without direct damage can decrease the risk perception [15,22]. A very remote perception of risk may mean less perceived need for information and it has been reported as a reason for low knowledge of the heat plan [23]. This perception could mean avoidant behavior towards preventive measures [17].

Locals also had a better knowledge about certain risk groups, symptoms and protective measures and knew better about the heat plan when compared with foreigners, despite having a lower education. This could be explained by locals being more exposed to local media and better targeted by local campaign messages [24]. This is also supported by our finding of greater heat plan knowledge in foreigners whose mother tongue was Portuguese or Spanish (in Lisbon or Madrid) compared to foreigners from countries speaking other languages. This can be explained by communication materials mostly being disseminated in Portuguese for Lisbon and in Spanish for Madrid [14], which may be less effective for residents of other nationalities. Evidence suggests that immigrants who are comfortable with the local language are more exposed to health information and have a higher trust in it than those who do not master the language [25]. The use of other languages for communication materials on heat should be piloted in neighborhoods with high proportions of foreigners. Individuals from other nationalities belonging to risk groups should be specifically addressed by their general practitioners in Lisbon and Madrid in order to enhance the recommendations to face extreme heat.

Similar surveys in Brussels and Amsterdam [18] identified a difference in knowledge among respondents with different educational levels where respondents with a lower educational level reported a lower knowledge. Consequently, it was recommended to target awareness-raising campaigns at citizens with a lower educational level in Brussels and Amsterdam. Our findings in Lisbon and Madrid show that educational level is not significantly associated with a lower knowledge about the plan. This difference between the study in Brussels and Amsterdam and our study may be due to education not being such an influencing factor on where citizens are more regularly exposed to extreme heat, such as in Lisbon and Madrid. We showed that citizens from other nationalities were less knowledgeable, independent of educational level, and should be specifically targeted. Both studies identified specific groups; therefore, they highlight the importance of addressing specific groups when communicating public health messages on heat.

The familiarity with the existing national heat plans was lower in Madrid (25.2%) than in Lisbon (37.2%), Amsterdam (33.1%) and Brussels (39.3%) [18]. When compared with other northern European cities such as London, Norwich, Brussels and Amsterdam [18,26], respondents in Lisbon and Madrid more often stated that their government provided too little awareness about extreme heat. This may be related to citizens in these two cities being less knowledgeable about the government’s actions, such as the heat plan, therefore feeling that their governments do not inform them enough. National and region-specific adapted campaigns about heat plans, specific groups and measures are therefore recommended [3]. Further qualitative methods could be used for in-depth understanding of the reasons for this knowledge.

The main limitation of our study is the small sample which doesn´t allow generalizability to the cities of Lisbon and Madrid. Nonetheless, these results provide insight on the knowledge and attitudes of citizens to health risks and heat plans. Another important limitation is that nationality was used as a proxy for the respondents’ mother tongue while that may not have been the case.

## 5. Conclusions

In Madrid and Lisbon, citizens’ knowledge of the impact of extreme heat was higher for practical concepts such as certain risk groups and protective measures than for the existence of a national heat plan. This practical knowledge can have public health implications, where the general population can be mindful of identifying those people at risk. Foreigners were less aware than locals, despite having a higher educational level. Knowledge of heat-related risk groups and how citizens can protect themselves from extreme heat should be improved. Tailored and targeted approaches should be considered during health campaigns to reach residents from other nationalities.

## Figures and Tables

**Table 1:** Demographic characteristics of the survey respondents, Lisbon and Madrid 2016.

Demographic Characteristics	Lisbon		Madrid		*p*-Value
	*n* = 129		*n* = 131		
	*n*	%	*n*	%	
Male sex	62	48.1	68	51.9	0.54
Age					
≤25 years old	21	16.3	27	20.6	0.49
26–65 years old	77	59.7	79	60.3	
>65 years old	31	24.0	25	19.1	
Educational level					
None or primary	17	13.2	25	19.2	0.32
High school or vocational training	52	40.3	54	41.5	
University degree	60	46.5	51	39.2	
Nationality					
Local (Portuguese or Spanish)	113	87.6	96	73.3	<0.01
Foreigners	16	12.4	35	26.7	

**Table 2:** Knowledge about extreme heat risk and policies, Lisbon and Madrid 2016.

Knowledge About Heat and Plan	Lisbon		Madrid		*p*-Value
	*n* = 129		*n* = 131		
	*n*	%	*n*	%	
Knowledge about the heat plan					
Yes or some idea	48	37.2	33	25.2	0.03
No	81	62.8	98	74.8	
Source of knowledge of the plan					
Television	38	79.2	17	80.9	0.98
Internet/social media	5	10.4	2	9.5	
Radio	5	10.4	2	9.5	
Knowledge of heat plan activation					
2015 (correct answer)	3	27.3	5	50	0.29
Other (wrong answer)	8	5.3	5	3.9	
Don’t know	37	62.7	22	37.3	
Knowledge of risk groups *					
Elderly	102	79.1	104	79.4	0.94
Children	52	40.3	77	58.8	<0.05
Babies	50	38.7	6	4.6	<0.01
Pregnant women	8	6.2	7	5.3	0.76
Medically-ill people	47	36.4	21	16.1	<0.01
Socially isolated people	3	2.3	1	0.7	0.30
Social problems	6	4.6	0	0.0	<0.05
Extreme physical effort	2	1.5	8	6.1	0.05
Obese people	6	4.6	8	6.1	0.60
Don’t know	4	3.1	9	6.9	0.15
Knowledge of symptoms *					
Headache	15	11.6	21	16.0	0.32
Sunburn	14	10.8	7	5.3	0.10
Fatigue	31	24.0	26	19.8	0.41
Sweating	20	15.5	15	11.5	0.35
Nausea	21	16.2	6	4.5	<0.01
Muscle cramps	1	0.7	0	0.0	0.31
Dehydration	52	40.3	34	25.9	0.01
Dizziness	7	5.4	40	30.5	<0.01
Low blood pressure	23	17.8	10	7.6	0.01
Don’t know	5	3.8	11	8.8	0.10
Knowledge of protective measures *					
Increased fluid intake	95	73.6	95	72.5	0.84
Avoid exposure to sun	83	64.3	49	37.4	<0.01
Keep windows closed	3	2.3	3	2.3	0.95
Adjust clothing	56	43.4	14	10.7	<0.01
Visit green areas	48	37.2	20	15.2	<0.01
Cool the body	13	10.8	15	11.4	0.9
Use fan or airconditioning	15	11.6	11	8.4	0.5
Avoid physical activity	6	4.6	3	2.3	0.18
Adjust diet	2	1.5	11	8.4	0.01
Use sunscreen	36	27.9	24	18.3	0.09
Use of sunglasses	4	3.1	6	4.6	0.05
Don’t know	0	0.0	8	6.2	<0.01

* Each response refers to a separate open question. The proportion of responses for each of the open questions is presented. *p*-values were calculated for each of the responses.

**Table 3:** Beliefs on personal heat susceptibility, increase in temperature and responsibility, Lisbon and Madrid 2016.

Beliefs on Heat Susceptibility, Increase in Temperature and Responsibility	Lisbon		Madrid		*p*-Value
	*n* = 129		*n* = 131		
	*n*	%	*n*	%	
Sensitive to heat					
Very much	47	36.4	43	32.8	0.20
Little or not	82	63.5	85	64.8	
Don’t know	0	0	3	2.3	
Sufficient awareness by government					
Too little	83	64.3	62	47.3	0.02
Adequate	42	32.5	62	47.3	
Too much	2	1.5	1	0.7	
Don’t know	2	1.5	6	4.6	
Believe increase in temperature					
No	29	22.5	17	13.1	0.02
Yes	86	66.7	106	81.5	
Don’t know	14	10.8	7	5.4	
Responsibility to take actions about increase in temperature *					
Government	92	71.3	80	62	0.11
Everybody	3	2.3	18	13.7	<0.01 **
Citizens	41	31.7	17	13.2	<0.01
Municipalities	46	35.6	5	3.8	<0.01
Private sector	31	24.0	2	1.5	<0.01 **
Don´t know	15	11.6	15	11.45	0.96

* Each response refers to a separate open question. The proportion of responses for each of the open questions is presented. We used Pearson Chi-square tests or Fisher’s exact test ** when appropriate. *p*-values were calculated for each of the responses.

**Table 4:** Age and educational level by nationality, Lisbon and Madrid 2016.

Age and Educational Level	Locals		Foreigners		*p*-Value
	*n* = 209		*n* = 51		
	*n*	%	*n*	%	
Age					
≤25 years old	35	16.7	13	25.5	<0.01
26–65 years old	118	56.5	38	74.5	
>65 years old	56	26.8	0	0	
Educational level					
None or primary	40	19.2	2	3.9	<0.01
High school or vocational training	77	37.0	29	56.9	
University degree	91	43.7	20	39.2	

## Appendix A. Questionnaire of the study (English version).

### **Introduction**

Good morning/afternoon. We are researchers from the Université catholique de Louvain, and we are trying to improve services with respect to extreme heat in Madrid. May I ask you some questions about this? This will take around 5 minutes.

Thank you for your time. This study is on the perception of the general public on extreme heat and the “National Heat Plan”, to find out to what extent people are familiar with it. This is important, also since there will be an increase in the number of heatwaves due to climate change. This study has been carried out in Lisbon, Brussels, Amsterdam, as part of a European project.

### **Questions**

(1) Do you live in Madrid?
  □ Yes
  □ No → If no, not eligible for this study!
(2) For how many years have you lived in Madrid?
(3) Are you aware of the existence of a “National Heat Plan” in which is activated if high temperatures are predicted for several days?
  □ Yes → go to question 4
  □ No, but I know that some measures exist → go to question 4
  □ No → go to question 6
(4) Where did you hear about this plan?
  □ Television
  □ Radio
  □ Newspaper
  □ Internet/social media
  □ Through relatives/friends
  □ In the street
  □ Other way, namely
(5) Do you know in which year the heat plan was activated the last time?
(6) Some people are more sensitive to extreme heat. Can you name some groups of people who have a higher risk for health effects due to extreme heat? (Don’t provide respondent with the options below! But please select the option(s) that best approximate the responder’s answer.)
  □ Elderly
  □ Newborns/babies
  □ Children
  □ People who take certain kind of medication/patients (medically-ill people)
  □ Obese
  □ Handicapped people or with mobility problems
  □ People who are socially isolated (homeless persons/migrants)
  □ People who perform a lot of physical efforts (sports/construction work)
  □ Other, namely
  □ I don’t know
(7) There are ways in which you can protect yourself from extreme heat. Do you know some things you could do to prevent health effects from extreme heat? (Don’t provide respondent with the options below! But please select the option(s) that best approximate the responder’s answer.)
  □ Increase consumption of fluids (water/soft drinks)
  □ Stay inside during the warmest times of the day
  □ Keep windows closed when the temperature outdoors is higher than indoors
  □ Adjust your clothing (light materials, light colours)
  □ Visiting green areas (forest, park, etc.)
  □ Cooling your body, e.g., by taking a shower, bath or swimming
  □ Using a fan or air-conditioning
  □ Avoid physical activity (sports)
  □ Other, namely
(8) Do you know which are the symptoms associated with the extreme heat? (Don’t provide respondent with the options below! But please select the option(s) that best approximate the responder’s answer).
  □ Headache
  □ Sunburn
  □ Fatigue
  □ Profuse sweating
  □ Nausea
  □ Muscle cramps
  □ Others
  □ I don’t know
(9) Do you consider yourself sensitive for extreme heat?
  □ Very much □ Somewhat □ Not at all □ I don’t know
(10) Do you think the government raises enough awareness for extreme heat?
  □ Too little □ Just enough □ Too much
(11) Do you think there is an increase in temperature in the last years?
  □ Yes □ No □ I don’t know
(12) Who do you think should be responsible to take measures against the increase in temperature?
  □ Government □ Madrid City hall □ Private sector □ Citizens □ I don’t know
(13) Do you have any other remarks/comments on extreme heat that might be of interest for us?
(14) Gender
  □ Male □ Female
(15) What is your age?
(16) What is your educational level?
  □ None
  □ Completed primary school
  □ Completed lower vocational education
  □ Completed general secondary education
  □ Completed secondary vocational education
  □ Completed senior general or pre-university education
  □ Completed college or university studies
(17) What is your nationality?

### **Closure**

Thank you very much for your participation. If you want to receive a short summary of our results, you can provide us with your email address. This will not be used for any other purpose.

Interviewer comments
